# Pathophysiology and histological anomalies in testicular tissues of *Calosoma olivieri* exposed to heavy metals generated by pesticide industries

**DOI:** 10.1038/s41598-026-41290-z

**Published:** 2026-03-23

**Authors:** Lamia M. El-Samad, Esraa A. Arafat, Hussein K. Hussein, Nessrin Kheirallah, Eman H. Radwan, Hala meshaal, Mohamed A. Hassan

**Affiliations:** 1 Department of Zoology, Faculty of Science, Alexandria University, Alexandria, Egypt; 2 Department of Zoology, Faculty of Science, Damanhour University, Damanhour, Egypt; 3 Protein Research Department, Genetic Engineering and Biotechnology Research Institute (GEBRI), City of Scientific Research and Technological Applications (SRTA-City), New Borg El-Arab City, Alexandria, 21934 Egypt

**Keywords:** Beetles, Bioindicator, *Calosoma olivieri*, Fertility impairment, Heavy metals, Industrial contamination, Oxidative stress, Pesticide industries, Testicular tissue, Ecology, Zoology, Environmental sciences, Biomarkers, Endocrinology

## Abstract

Recently, there has been a critical demand for continuous investigations into environmental contamination with heavy metals to evaluate their detrimental impacts on different species inhabiting these contaminated areas. Therefore, we used *Calosoma olivieri* (Coleoptera, Carabidae) in the present study as a bioindicator for investigating pollution with heavy metals generated by pesticide industries in Kafr El-Zayat, Egypt. Toward this end, we probed the pathophysiological, histopathological, and ultrastructure anomalies of *C. olivieri* testicular tissues from the contaminated area in comparison to the clean site. Evaluation of heavy metal accumulation within testicular tissues was conducted employing energy-dispersive X-ray spectroscopy analysis. Furthermore, various biochemical parameters were measured to evaluate oxidative stress and the detoxification state of *C. olivieri*. Notably, significant inhibitions in antioxidant and detoxifying enzymes, including superoxide dismutase, catalase, glutathione S-transferase, glutathione peroxidase, reduced glutathione, and glutathione reductase, combined with elevated lipid peroxidation and protein carbonyl content were detected in testicular tissue of *C. olivieri* obtained from a polluted area due to the accumulation of multiple heavy metals. Additionally, severe structural aberrations were observed in the testicular tissues of *C. olivieri* obtained from the contaminated site compared to the control, including mitochondrial and nuclear destruction and deformed spermatogenic elements, along with obvious signs of tissue necrosis. Collectively, these results evinced impairment of male testicular tissues in beetles from the polluted location, which could serve as a reliable indicator of heavy-metal pollution in industrial areas.

## Introduction

Anthropogenic heavy metal pollution arising from different activities, including sewage irrigation, mining, electroplating, and industrial operations, represents a major global environmental concern^1–3^. Consequently, both human health and ecological integrity are imperiled by the release of heavy metals, even at low concentrations^4,5^. Owing to their deleterious impacts and persistence, the US Environmental Protection Agency has classified several heavy metals, including chromium, lead, cadmium, and arsenic, as hazardous pollutants^6^. Given the extensive discharge of heavy metals from industrial operations, the surrounding environments are predisposed to the accumulation of these pollutants in soil, making human exposure is inevitable^7–9^.

Among different industrial activities, the production and extensive application of pesticides, including fungicides and insecticides, have emerged as the most prevalent source of anthropogenic heavy metal pollution^10,11^. Due to rapid population growth, there is a high demand to enhance the food production and to sustain agricultural crops against various pests^12^. This entails a substantial rise in pesticide manufacturing, engendering the emancipation of toxic compounds into the ecosystem^11^. Recent studies reported that about 2 million tons of pesticides are exploited globally, comprising about 47.5% herbicides, 29.5% insecticides, 17.5% fungicides, and 5.5% other pesticide types^11,13^.

It is widely recognized that Egypt is an agriculture-based country due to the Nile River and the abundance of fertile land along its banks^14,15^. Therefore, various chemicals are widely utilized across a wide range of industrial sectors, particularly those involved in pesticide production^15^. It has been reported that approximately 50% of all industrial activities in Egypt are concentrated in Greater Cairo and around 40% in Alexandria, while the remaining industries are distributed throughout the Delta Region and Upper Egypt^15^. Among the industrial cities in Egypt, Kafr El-Zayat located in Al Gharbiyah Governorate in the western Nile Delta, is recognized as both an industrial and agricultural hub. It hosts several industrial companies, including the El-Mobidat Company, which produces over 40 chemical products used in agricultural and domestic fertilizers and pesticides. Additionally, the El-Malyia Company produces pesticides and industrial chemicals, such as superphosphate, multiple forms of sodium fluosilicate, sulfuric acid, and ferrous sulfates. Besides, The Salt and Soda Company manufactures a wide range of products, including soap, animal feed, wax, liquid and solid sodium silicates, oils, and other products^16^. Thus, the discharge of toxic compounds, including heavy metals, into the surrounding environment, particularly during pesticide manufacturing, is highly predictable. In this context, a previous assessment of heavy metals accumulation in the agricultural soils of Kafr El-Zayat employing laser ablation inductively coupled plasma mass spectrometry (LA-ICP MS) and inductively coupled plasma optical emission spectroscopy (ICP-OES) demonstrated the incidence of Co, Cr, V, Zn, Pb, Ni, Mn, and Cu. Critically, the concentrations of Cr, Ni, and V in the soil surpassed the threshold limits approved by the Canadian Soil Quality Guidelines^17^.

Several studies demonstrated that prolonged exposure to heavy metals may result in severe cellular impairment and functional aberrations in various organisms^18–21^. It is believed that heavy metals translocate across various biological membrane barriers, penetrating cells either through specialized transporters or non-specific chemiosmotic metal uptake mechanisms^1,22^. This provokes the denaturation and inactivation of enzymes by displacing native metal cofactors at the enzyme active sites^1,23,24^. Besides, intracellular heavy metals can trigger DNA impairment by reducing DNA content, compromising structural integrity, and inducing random mutations^1,25,26^. To assess and monitor both anthropogenic and geogenic soil pollution, bioindicators are broadly employed as a cost-effective and reliable approach^11^. Effective bioindicators should meet several prerequisites, including wide geographical distribution, well-established taxonomical and ecological information, high susceptibility to environmental alterations, and ease of collection and sampling^27^.

Among potential bioindicators, various insect genera significantly contribute to assessing environmental health owing to their distinctive structural and functional organization, which underlies complex morphology and advanced physiological processes^28,29^. Additionally, insects possess various sense organs and reveal complex behavioral properties, making them highly vulnerable to environmental pollutants^30^. Therefore, terrestrial insects, particularly beetles (Coleoptera, Insecta), are widely exploited as bioindicators for multiple ecological pollutants. Their broad distribution, short life cycle, and organ sensitivity, especially of the reproductive system, facilitate the detection of physiological and histological aberrations induced by environmental toxins^31,32^. It is well established that insects can activate specialized physiological and biochemical detoxification mechanisms to alleviate the pernicious effects of heavy metal exposure and sustain redox homeostasis^33^. These detoxification mechanisms predominantly involve vital oxidative defense enzymes, such as glutathione S-transferase (GST), superoxide dismutase (SOD), catalase (CAT), and ascorbate peroxidase (APOX)^18^. These antioxidant enzymes significantly contribute to counteracting oxidative impairment and serve as biomarkers for evaluating oxidative stress^34^. Critically, the production of surplus reactive oxygen species (ROS) compromises antioxidant defense systems, resulting in oxidative impairment^12^.

In light of the aforementioned information, the current study provides a novel and integrative evaluation of heavy metal contamination originating from pesticides-manufacturing industries in Kafr El-Zayat, Al Gharbiya Governorate, Egypt. To the best of our knowledge, only one previous investigation assessed heavy metal levels in agricultural soils of Kafr El-Zayat, relying solely on chemical approaches and has not considered the deleterious effects of these contaminants on biological organisms. Therefore, in this study, we utilized *Calosoma olivieri* collected from areas surrounding pesticides-manufacturing factories in Kafr El-Zayat, Al Gharbiya Governorate, Egypt as a bioindicator species due to its predominance in the study area. We specifically probed its testicular tissues, a highly sensitive organ to environmental toxicants, to explore the potential detrimental influences of discharged heavy metals on reproductive organs of terrestrial insects and other organisms accordingly. The hazardous effects of these heavy metals on testicular tissues were evaluated by quantifying their accumulation. Besides, pathophysiological alterations were investigated by evaluating various oxidative stress biomarkers in testes compared to control testes obtained from insects collected from uncontaminated site. Moreover, histopathological and ultrastructure attributes of *C. olivieri* testicular tissues following heavy metal exposure were inspected in relation to those of control tissues.

## Materials and methods

### Study areas

In the present study, two distinct geographical locations were selected for the collection of the bioindicator species *Calosoma olivieri*. The first site, designated as site A, served as the control and was previously identified as a non-polluted site^35^, which is situated within the garden of the Faculty of Science, Alexandria, Egypt (longitude: 29°55′9.13" E; latitude: 31°12′25.60" N). Conversely, the second location, designated as site B, was selected to represent a polluted environment located in Kafr El-Zayat, Al Gharbiya Governorate of Egypt (longitude: 30°48′43.14" E; latitude: 30°50′51.98" N). It is recognized that Kafr El-Zayat is a densely populated urban area that hosts a wide array of industrial operations, including Kafr El-Zayat (KZ) for pesticides and chemicals company. Therefore, sampling at site B focused on areas with intensive chemical and fertilizer manufacturing activities, particularly in proximity to the KZ Company, which manufactures various pesticides and chemicals.

### Samples collection

A total of 320 adult beetles (mean weight of 1.96 g) were randomly collected from the study locations (sites A and B) to serve as bioindicators. Beetles were collected in August 2021 during the early morning hours of the summer season in Egypt. The insects were instantly transferred to the entomology lab before being identified at the Entomology Department, Faculty of Agriculture, Alexandria University, Alexandria, Egypt, as *Calosoma olivieri*, Carabidae. The beetles, *C. olivieri*, were maintained under regulated laboratory conditions at 25 ± 2 °C and 85% humidity with a 12:12 h light/dark for 24 h before commencing the dissection process to mimic their natural environmental habitat. In this study, a total of 160 male *C. olivieri* beetles were chosen and dissected through opening their abdominal cavities to harvest testicular tissues. For biochemical analyses, testes were immediately preserved at − 80 °C for the respective assays. For histological and ultrastructure investigations, beetles were injected with 0.02 ml of a fixative mixture consisting of 4% formaldehyde and 1% glutaraldehyde (4F:1G) at pH 7.2, and their testicular tissues were then collected through dissection as previously explained^36^.

### Heavy metals assessment in testicular tissues of *C. olivieri*

To quantify heavy metal accumulation in beetle testes, three individuals from each site were selected, and their testicular tissues were analyzed to determine the metal content. This examination was mainly carried out to verify the potential accumulation of heavy metals released from pesticide-manufacturing facilities at the site B in the testes prior to assessing their detrimental effects on the tissues. The sections of the dissected testicular tissues underwent microelemental analysis with the help of energy-dispersive X-ray spectroscopy (EDX) coupled with a scanning electron microscope (Jeol JSM-5300, Japan). Consequently, testicular tissues were visualized, and elemental peaks were automatically identified through integrated SEM–EDX software. The SEM–EDX analysis provides visualization of tissues and quantitative determination of metal elements, detecting a wide range of element with high sensitivity.

### Biochemical analysis

Various biochemical parameters were measured to evaluate the oxidative stress status in *C. olivieri* testicular tissues from each site. Therefore, dissected testicular tissues from fifteen insects obtained from each site were utilized to prepare testis homogenates following previously reported procedures^37^. Briefly, testes were weighed and homogenized for 30 s in a phosphate buffer (pH 7.0), followed by clarification at 4 °C and 10000 × g for 30 min to obtain supernatants, which were then kept at − 80 °C until further examinations.

Superoxide dismutase (SOD) activity in the testicular tissue of *C. olivieri* was measured following the procedures of Misra and Fridovich^38^. On the other hand, the activities of glutathione peroxidase (GPx) and reduced glutathione (GSH) were assessed employing the method of Cichoski et al.^39^ and Beutler et al.^40^, respectively. Additionally, catalase (CAT) and glutathione S-transferase (GST) activities, along with malondialdehyde (MDA) levels, were determined as previously reported^41–43^. Furthermore, glutathione reductase (GR) activity in testicular tissues was evaluated utilizing a commercial colorimetric kit (GR kit, Nanjing Jiancheng Bioengineering Inc., Nanjing, China), whereas protein carbonyl was assessed employing a colorimetric assay kit (Cat. No. 10005020, Cayman Chemical, Solana Beach, CA, USA).

### Histopathological and ultrastructural examination

Testes of *C. olivieri* collected from sites A and B were fixed in 4F:1G buffer and then postfixed in 2% OsO_4_ solution for 2 h at 4 °C as reported earlier^44^. Following this, tissues were dehydrated in ascending ethanol grades and then embedded in an Epon-Araldite resin mixture (Sigma Aldrich, France). Next, employing an ultramicrotome (LKB Bromma 2088 Ultrotome, Leica Instruments, USA), testicular tissues were sectioned at a thickness of 0.5 μm, mounted on glass slides, and then stained with toluidine blue for semithin investigation. The sections were inspected employing a light microscope (Olympus CX31, Japan).

For ultrastructural inspection, ultrathin sections of 60 nm thickness were obtained and mounted on 200-mesh naked copper grids. The ultrastructure features were surveyed by staining with uranyl acetate and lead citrate, and then probed using a transmission electron microscope (TEM, Joel 100 CX, Japan) operated at an acceleration voltage of 80 kV^45^.

### Statistical analysis

All investigations presented in the current study were executed in three replicates. Statistical analysis was performed employing GraphPad Prism (Version 8, GraphPad Software Inc., USA). Significant differences between control beetles and those collected from the contaminated site were evaluated using a two-tailed unpaired Student’s *t*-test. Statistical significance was considered at *p* ≤ 0.05. All the graphs plotted in this study show the data as the mean ± SD.

## Results

### Determination of heavy metal accumulation in testes of *C. olivieri*

**Fig. 1:**
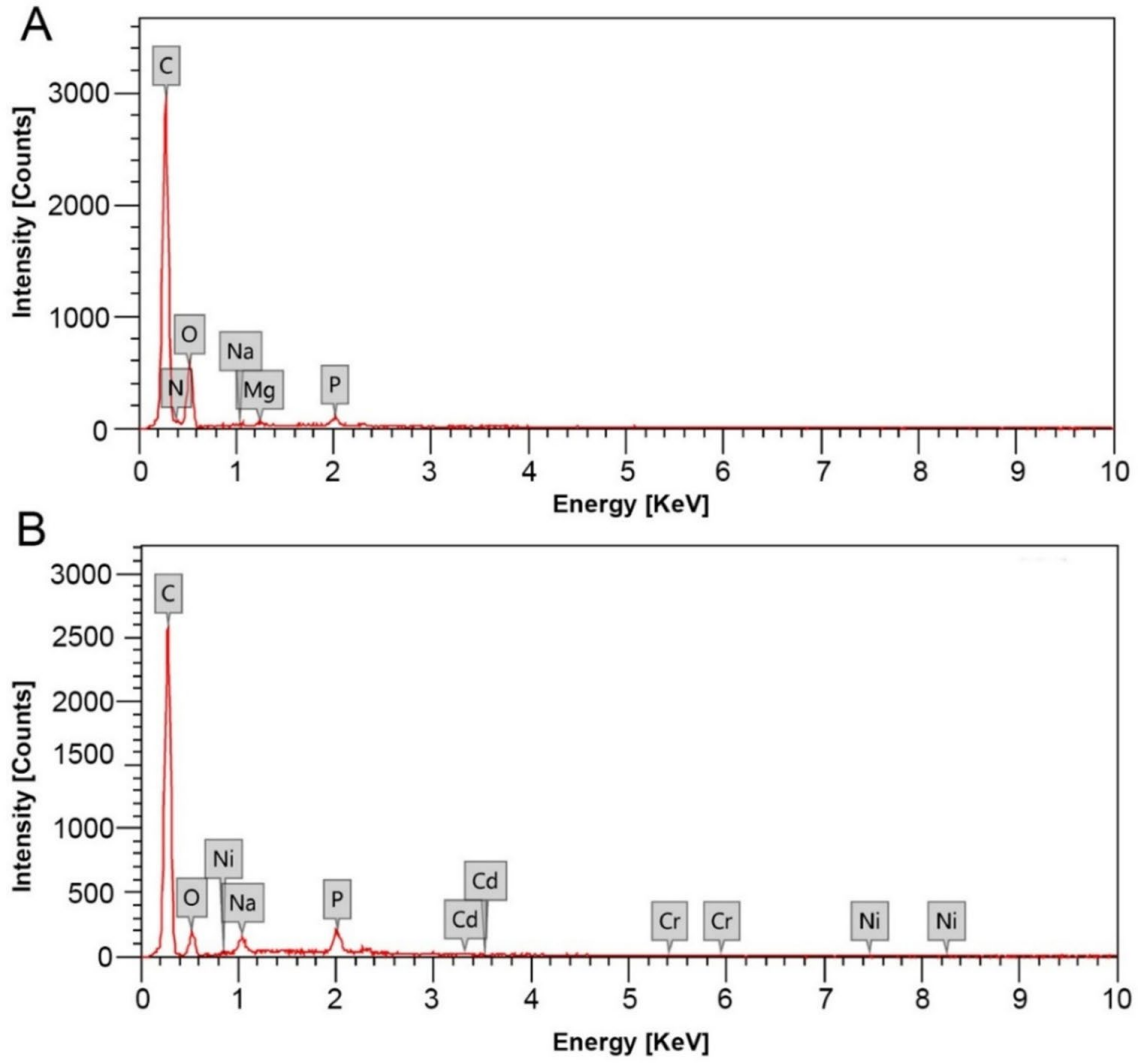
EDX spectra of (**A**) *C. olivieri* testes collected from the control location A and (**B**) *C. olivieri* testes from the polluted location B.

EDX analysis was used to quantify the concentrations of elements, including hazardous heavy metals, accumulated in the testicular tissues of *C. olivieri* obtained from the clean site (A) and contaminated site (B). EDX assessment of *C. olivieri* testicular tissues collected from the uncontaminated site (A) showed only essential elements, since this site is remote from industrial activity and relies solely on organic fertilizers, thereby precluding the accumulation of heavy metals as illustrated in Fig. 1. By contrast, three heavy metals, including Cr, Ni, and Cd, were detected in the testes of *C. olivieri* sampled from the contaminated area, which includes pesticide industries. Among these heavy metals, Ni reported the highest ratio of 0.32 ± 0.1%, while Cd and Cr concentrations reported at 0.16 ± 0.05% and 0.06 ± 0.01%, respectively, as presented in Table 1. Additionally, it is apparent from the data that there were marked reductions in concentrations of C, O, Na, and P in the testicular tissues of *C. olivieri* from the polluted area (B) in relation to those from the control area (A). Moreover, N and Mg were undetectable in *C. olivieri* testicular tissues from site B.

**Table 1:** EDX analysis for the testicular tissues from site A and B (control and polluted sites, respectively).

Element	Control site (Site A)	Polluted site (Site B)
	Mass (%)	Mass (%)
C	68.29 ± 0.27^a^	81.60 ± 0.34^b^
N	2.35 ± 0.34^a^	–
O	27.45 ± 0.45^a^	12.62 ± 0.36^b^
Na	0.27 ± 0.04^a^	1.69 ± 0.09^b^
Mg	0.39 ± 0.04^a^	–
P	1.26 ± 0.07^a^	3.65 ± 0.12^b^
Cr	–	0.06 ± 0.01^b^
Ni	–	0.32 ± 0.1^b^
Cd	–	0.16 ± 0.05^b^

Data are depicted as mean ± SD (n = 3). Different letters indicate the significant differences in the mass of elements in testicular tissues *C. olivieri* sampled from the two locations (sites A and B) at *p* < 0.05. The symbol (—) denotes elements that were not detected in the testicular tissues.

### Biochemical results

**Fig. 2:**
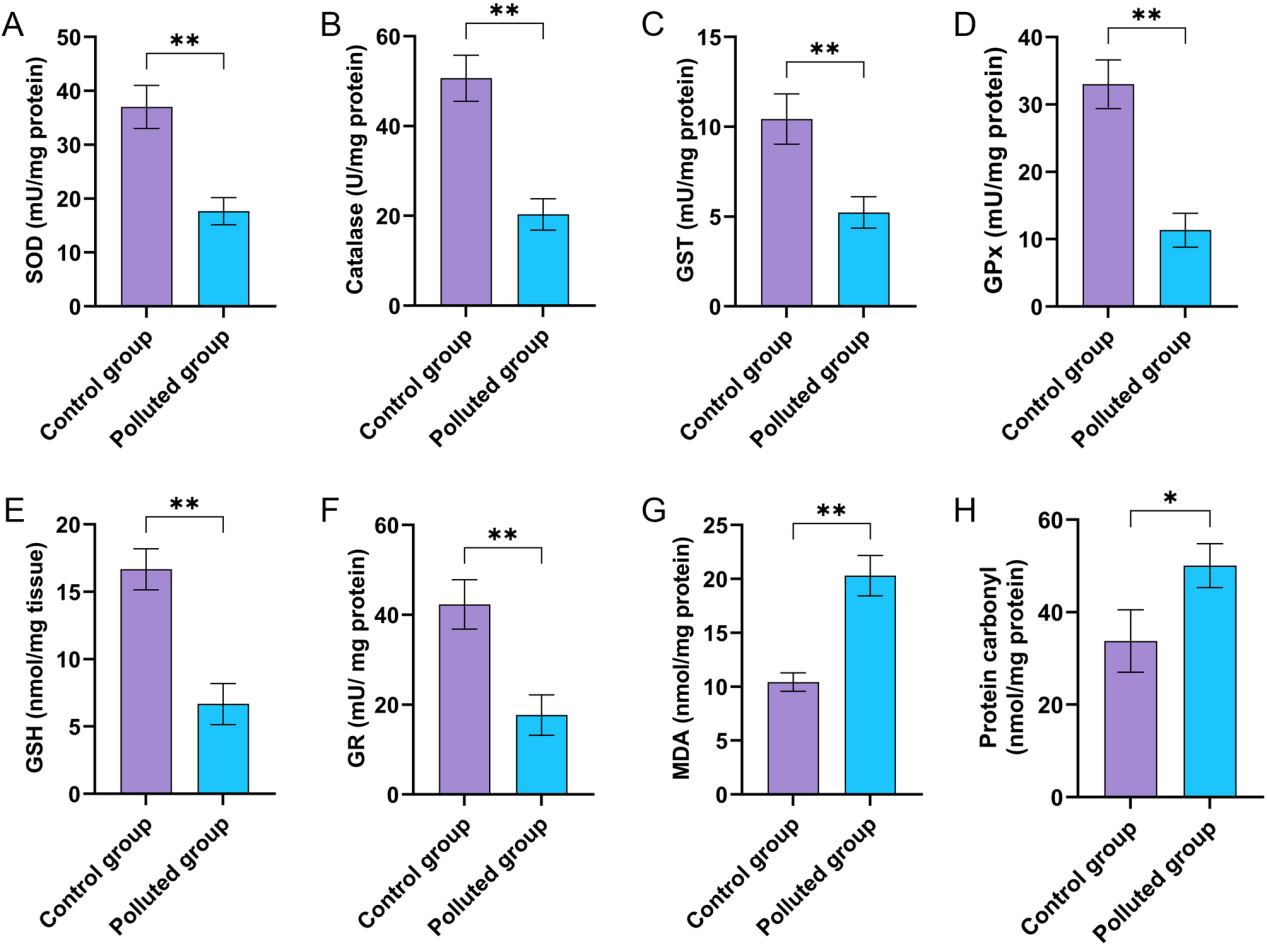
Oxidative stress parameters assessed in *C. olivieri* testicular tissues from the contaminated site (**B**) in relation to the reference site (**A**). Evaluation of (**A**) superoxide dismutase (SOD) activity, (**B**) catalase activity, (**C**) glutathione S-transferase (GST) activity, (**D**) glutathione peroxidase (GPx) activity, (**E**) reduced glutathione (GSH) level, (**F**) glutathione reductase (GR) activity, (**G**) malondialdehyde (MDA) level, and (**H**) protein carbonyl content. The figures are plotted as mean ± SD, showing significant differences between beetles collected from reference and contaminated sites (**p < 0.01 and *p < 0.05).

In order to assess cellular redox homeostasis in testicular tissues of *C. olivieri* from reference and polluted sites, various oxidative stress parameters were measured as depicted in Fig. 2. It can be extrapolated from these findings that there are noticeable aberrations in antioxidant and detoxification parameters in testicular tissues from the polluted site. Beetles from the contaminated location exhibited a significant diminution in the activities of antioxidant enzymes, including SOD, CAT, GST, GPx, and GR in relation to those collected from the reference site. Additionally, the GSH level in the testes from the contaminated area was substantially lower than that observed in control beetles. Furthermore, lipid peroxidation analysis revealed a substantial elevation of MDA in the testes of beetles from polluted beetles compared to control tissues. Moreover, protein carbonyl content was also significantly augmented in the testes of polluted beetles in relation to that from the control testes. Altogether, it can be inferred from these findings that heavy metal agglomeration in beetles’ testes impaired the antioxidant defense system, thereby compromising detoxification capacity and cellular redox homeostasis.

### Histopathological investigation of testes from *C. olivieri*

**Fig. 3:**
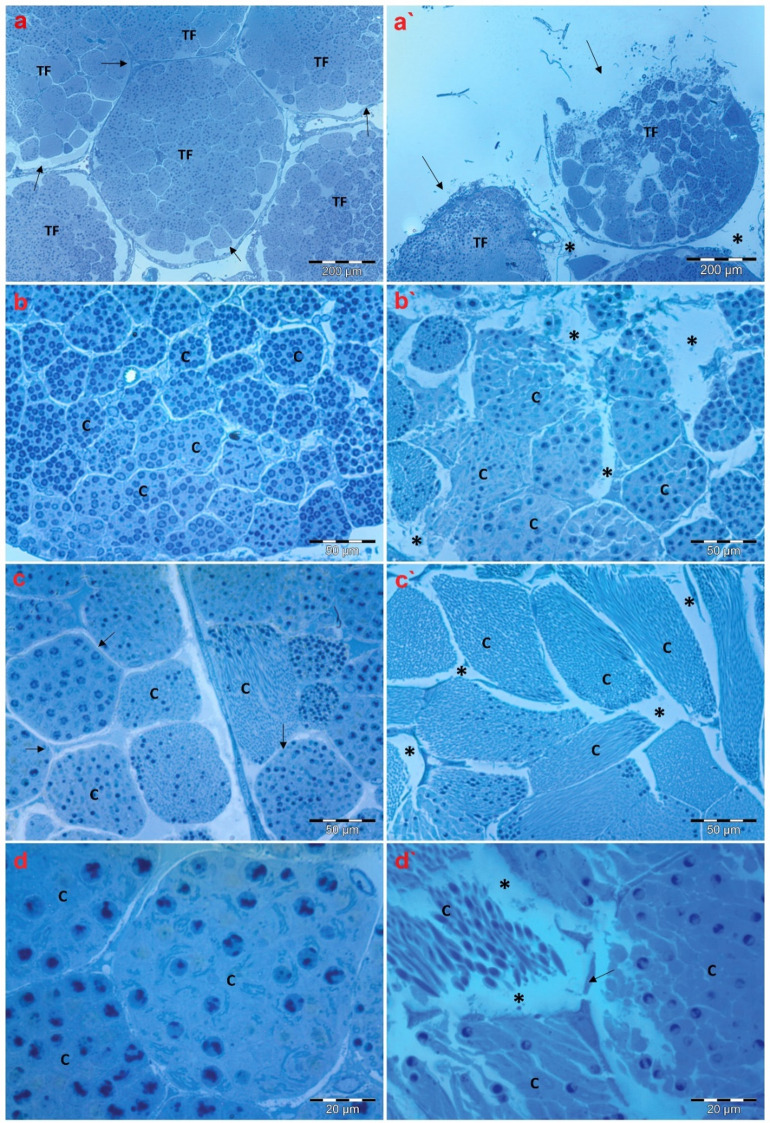
Histological structures of *C. olivieri* testicular tissues collected from reference and polluted locations. Fig. (**a–d**) demonstrate the typical structure of testes dissected from the reference group, showing (**a**) testicular follicles (TF) with intact follicular walls (arrows), (**b**) several cysts (C) within the testicular follicle, (**c**) the normal walls of the cysts (arrows), and (**d**) cysts (**C**) filled with various spermatogenic elements. Fig. (**a**`–**d**`) depict the alterations in histological attributes of testes dissected from the beetles collected from the contaminated location, demonstrating (**a**`) rupture of the follicular walls (TF) (arrows) with noticeable separation between each TF (asterisks), (**b**`) cysts with necrotic signs (asterisks), (**c**`) vacuolation (asterisks) between the cysts, and (**d**`) damaged cyst walls (arrow), slits, and vacuolation within the cysts (asterisk). (**a** and **a**`) (scale bar = 200 µm). Fig. (**b**, **b**`, **c**, and **c**`) (scale bar = 50 µm). Fig. (**d** and **d**`) (scale bar = 20 µm).

Histopathological features of testicular tissues from the beetles, *C. olivieri*, collected from the clean spot (A) and the contaminated area (B) were probed as delineated in Fig. 3. Semithin analysis of the control testes demonstrated the regular structure of testicular tissues, including numerous testicular follicles (TF), which consist of several cysts (C) with different spermatogenic elements as delineated in Fig. 3a. Additionally, Fig. 3b depicts that the cysts within the TF showed the typical organization of spermatogenic cells with different division stages. Besides, inspection of the cysts showed normal cyst walls, and the spaces between the cysts were almost equal between all cysts as displayed in Fig. 3c and d. Moreover, semithin images revealed no anomalies, such as vacuoles or slits, within the cysts.

Conversely, several deformities could be noticed in the testes of *C. olivieri* from the contaminated location. Furthermore, obvious damage was observed in the TF as indicated by ruptured walls and noticeable separation between each TF, reflecting impairment in the connective tissue, which holds the TF together as shown in Fig. 3a`. Detailed examination of the TF revealed the damage at the cyst level as diverse cysts emerged with necrotic signs combined with abnormal tissue appearance compared to the control group from site A (Fig. 3b`). Furthermore, impaired cyst walls were detected along the TF, with signs of vacuolation as portrayed in Fig. 3c`. Upon investigating the sections at high magnification, damaged cyst walls, slits, and vacuolation within the cysts were readily recognized as demonstrated in Fig. 3d`.

### Ultrastructure analysis of testes of *C. olivieri*

**Fig. 4:**
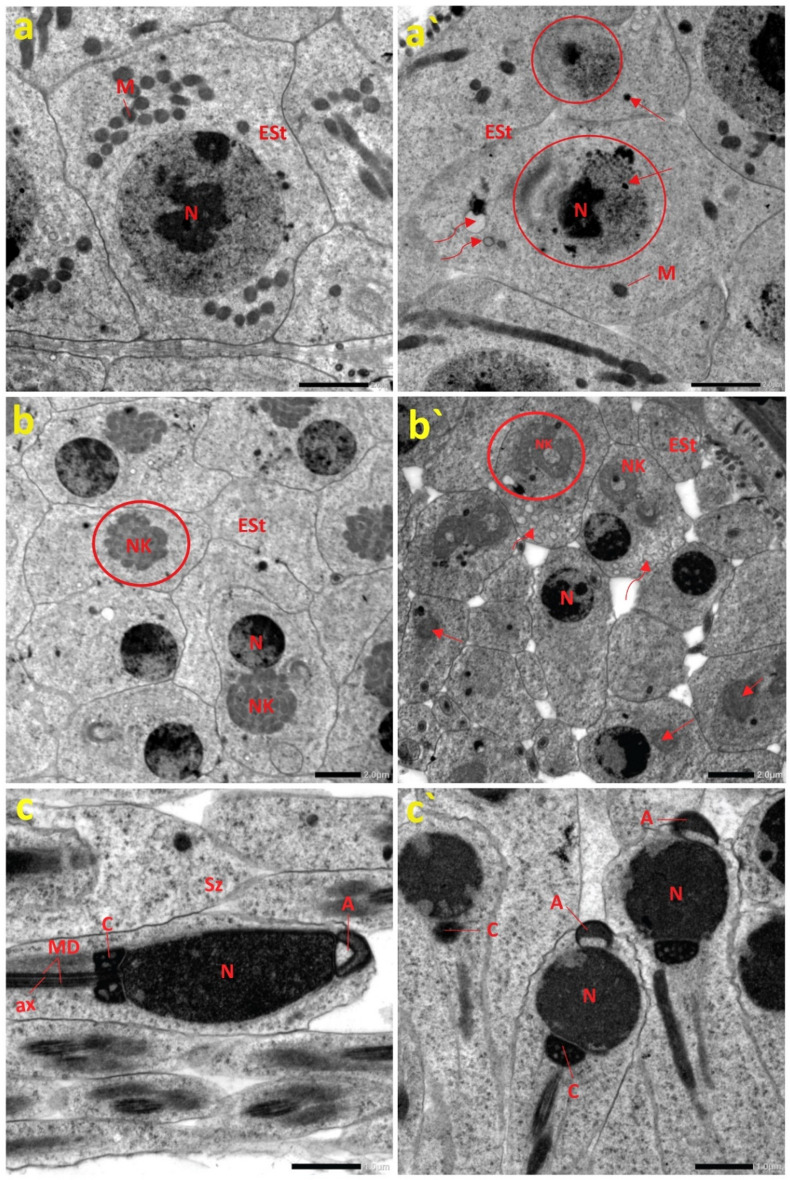
Ultrastructural analyses of *C. olivieri* testes taken from reference and polluted sites. Fig. (**a**–**c**) portray the ultrastructure of the spermatogenic elements of the reference group, showing (**a**) the early spermatids (Est) appear typically with aggregated mitochondria (M) around a large spherical nucleus (N), (**b**) the typical structure of mitochondrial Nebenkern (NK) found in the early spermatids (Est), (**c**) the spermatozoa structure, acrosome (A), axoneme (ax), and centriole (C), along with mitochondrial derivatives (MD). Fig. (**a**`–**c**`) illustrate the ultrastructure of the spermatogenic elements of the beetles from the polluted site, revealing (**a**`) the early spermatids (Est) with the nonobvious nuclear envelope (circle) and vacuoles within the cytoplasm (curved arrows), (**b**`) disintegration of the mitochondrial Nebenkern (NK) (circle), degeneration of (NK) (arrows), and vacuolation (curved arrows), and (**c**`) the aberrations in spermatozoa structural, including the abnormal chromatin condensation within nuclei (N). Fig. (**a**, **a**`, **b**, and **b**`) (scale bar = 2.0 µm). Fig. (**c** and **c**`) (scale bar = 1.0 µm).

To accurately determine the extent of deformation in the testes from the impacted location in relation to those from the reference site, we surveyed the ultrastructural features of *C. olivieri* testes employing TEM. Electron micrograph analyses of the testicular tissues from site A revealed typical structures of the spermatogenic elements, including early spermatids (Est) that appeared in a normal shape with aggregated mitochondria (M) around a large spherical nucleus to further generate mitochondrial Nebenkerns (NK) as presented in Fig. 4a and b. In addition, normal spermatozoa were observed with a triangular acrosome (A) in front of an oval head, followed by a centriole and a long tail, consisting of a central axoneme (ax) and two mitochondrial derivatives (MD) around it (Fig. 4c).

By contrast, several anomalies could be discerned in the spermatogenic elements of testes from site B. It is perceived in Fig. 4a` the emergence of anomalous early spermatids, as they were found with fewer mitochondria around the nucleus, combined with an indistinct nuclear envelope and vacuoles within the cytoplasm. Besides, the degenerated mitochondrial Nebenkern (NK) can be observed as illustrated in Fig. 4b`. Furthermore, spermatozoa with abnormal acrosomes (A) and abnormal chromatin condensation within the nuclei were detectable as depicted in Fig. 4c`. We recapitulated all anomalies detected in *C. olivieri* testicular tissues obtained from the polluted area (site B) as presented in Table 2.

**Table 2:** Recapitulation of the abnormalities detected in the testicular tissues of *C. olivieri* from the polluted site (site B).

Structure	Histological and ultrastructural anomalies
Testicular follicles (TF)	Ruptured walls of TF Noticeable separation between each TF Damaged cysts walls Vacuolation
Early spermatids (Est)	Disintegrated mitochondrial Nebenkern (NK) Nonobvious nuclear envelope Vacuoles
Spermatozoa	Abnormal acrosome (A) Abnormal chromatin condensation

## Discussion

Recently, substantial consideration has been focused on evaluating heavy metal accumulation in specific geographical regions, particularly with the industrial expansion, using various organisms as bioindicators and assessing their pernicious effects on these indicators due to unavoidable exposure of different organisms and human populations^46–48^. Due to the facile collection of terrestrial insects from contaminated sites and their direct exposure to various pollutants, they could serve as ideal bioindicators for assessing the deleterious impacts of heavy metals accumulated in different tissues^28^. Among organs sensitive to pollutants, reproductive organs are widely recognized as particularly vulnerable to the adverse consequences of heavy metals, even at low accumulation levels^20,49^. In the present study, we employed adult male *C. olivieri* collected from industrial regions in Kafr El-Zayat, Al Gharbiya Governorate of Egypt (Site B) as a model species due to its prevalence in the study area to appraise heavy metals accumulated in testicular tissues and the associated harmful effects compared to wild beetles collected from a non-contaminated reference site (Site A). This could provide a profound comprehension of the pollution status of site B and highlight the potential damaging impacts on organism and human beings residing this site, particularly workers residing or operating within the study area.

As anticipated, the EDX analysis of *C. olivieri* testicular tissues from the uncontaminated site (A) revealed only indispensable elements since the garden of this site is situated far from any industrial operations and its plants are fostered exclusively with organic fertilizers, thereby preventing the release of heavy metals into the surrounding agricultural soils. Consequently, the possibilities of these hazardous metals accumulation in terrestrial insects examined in this study are negligible. Conversely, the results revealed the agglomeration of Ni, Cd, and Cr in the testes of *C. olivieri* collected from the polluted site adjacent to pesticide-manufacturing facilities. Previous studies reported that pesticide-manufacturing industries consider one of the major sources of heavy metal discharge into soil and water^50^. Previous investigations reported that analyses of various pesticide formulations showed the prevalence of heavy metals, either as active ingredients or as impurities^51^. Notably, the most commonly detected heavy metals in pesticide active ingredients include Cu, As, Pb, Hg, Cr, Zn, Al, Li, Ba, B, and Ti^51,52^. Additionally, investigations of pesticides utilized for pest management in Japan revealed the occurrence of Cd, Hg, As, Cu, Zn, and Pb as contaminant impurities^53^. Besides, earlier investigations employing inductively coupled plasma/optical emission spectrometry (ICP-OES) analysis and ICP mass spectrometry demonstrated the incidence of As, Cr, Co, Pb, Cu, Tl, and Ni as contaminants in several pesticides^10,54^. It is proposed that certain heavy metals pollute pesticides throughout the manufacturing process, whereas others are deliberately incorporated into the form of nanopesticides to boost the efficacy of pesticides^10,55^. Another research conducted on honeybees (*Apis mellifera ligustica*) collected from several locations in the Umbria area of central Italy manifested the predominance of Cd, Cu, Mn, and Zn in bees, which is likely associated with the utilization of pesticides and fertilizers that contribute to soil pollution^56^. It is worth noting that the prevalence and concentrations of heavy metals vary substantially among different pesticides; therefore, pesticides manufacturing can discharge different types of heavy metals into the surrounding environment^51^.

Our findings demonstrated that the accumulation of these metals was accompanied by deregulation of different essential elements required for metabolic pathways, including N, O, and Mg. This disorder is likely attributable to the hazardous effects of heavy metals^57^, which may diminish or preclude the absorption of essential elements. The observed deficiency of Mg in testes from the polluted site implies impaired male fertility due to the key role of Mg in spermatogenesis and sperm motility, since the adenosine triphosphate (ATP) and cyclic adenosine monophosphate (cAMP) syntheses necessitated for sperm motility are a magnesium-dependent processes^58–61^. Moreover, the notable reduction in oxygen level in testes of beetles exposed to heavy metals further reinforces this explanation, as oxygen is imperative for ATP synthesis, in addition to its particular function in sustaining self-renewal and differentiation capacities of spermatogonial stem cells^62,63^. Similarly, the lack of N in contaminated testes further evidence the deleterious effects of heavy metals on testicular tissues of *C. olivieri*, given the pivotal role of nitrogen-containing compounds in male fertility, including sperm function and the safeguarding of the integrity of blood-testis barrier^64,65^. Previous studies demonstrated that Cd accumulation impaired the absorption of Zn, Ca, and Cu into the bloodstream and different tissues, leading to the low dietary intake of these vital elements^18^. Importantly, the diminution of indispensable elements may result in their displacement from the regular binding site. This may engender the aberrant interaction between heavy metals and key proteins and enzymes implicated in the modulation of biochemical pathways, causing cellular malfunction and ultimately toxicity^20,66^. On the other hand, the significant increase in phosphorus (P) in polluted testicular tissues suggests potential reproductive impairment. This result is in agreement with earlier investigations, which reported that increased phosphorus levels incited oxidative stress and inflammation, along with their correlation with erectile dysfunction^67,68^.

It is also established that heavy metal accumulation within insect tissues stirs up ROS overproduction, thereby instigating a cascade of dysregulations in physiological biomarkers^37^. Accordingly, we assessed different antioxidants in testicular homogenates of *C. olivieri*, demonstrating pronounced disorder in the activities of SOD, CAT, GPx, GR, and GST, along with marked aberrations in GSH, MDA, and protein carbonyl levels in testicular tissues of *C. olivieri* collected from the contaminated location, in comparison to those from the unpolluted location. These outcomes are likely related to the disruption of the antioxidant system, particularly through the direct interaction of heavy metals with sulfhydryl groups of antioxidant enzymes, including SOD, GPx, and GR^20^.

Of particular interest, Ni accumulated within *C. olivieri* testicular tissues can directly bind to DNA and induce ROS production through nickel-catalyzed redox reactions^69^. Furthermore, previous research reported that CAT depletion may be linked to the strong affinity of Ni for histidine residues, which are crucial for CAT activity^69^. In a similar way, cellular antioxidant properties can be adversely impacted by Cr, leading to oxidative stress, while Cd exerts a detrimental influence on testicular tissue through interactions with biomolecules, such as DNA, lipids, and proteins. Previous studies postulated that the interaction between Cd and proteins is predominantly mediated by binding to thiol groups (-SH), resulting in protein inactivation and consequent disorder of redox homeostasis^18^. Critically, malfunctions in SOD and GSH activities may be explained by their strong binding to Cd, forming complexes of Cd-SOD and Cd-GSH that alter their conformational structures^18^.

Another mechanism of heavy metal toxicity relies on eliciting surplus ROS, which indirectly foments oxidative stress impairment in cells^70^. Among the principal consequences of oxidative stress, lipid peroxidation is prominent due to the high affinity and interaction of ROS with polyunsaturated fatty acids. It is well known that MDA is a by-product biomarker of lipid peroxidation because of its ability to interact with DNA and proteins^69^, inciting DNA impairment and altering protein structure^71^. Accordingly, MDA proliferation in the current study signifies ROS overproduction and may represent a major genotoxic agent in the testicular tissue of contaminated *C. olivieri.* This explanation concurs with prior investigations, which showed increased MDA in *Sinopotamon henanense* testicular tissue induced by lead exposure^72^. The rise in MDA in the current study corroborated augmented lipid peroxidation, which was accompanied by increased protein carbonyl level as a consequence of of ROS overflow. This could be elucidated by the strong correlation between lipid peroxidation and protein carbonylation, since reactive carbonyl species can be generated by lipid peroxidation^73,74^.

Conversely, the noticeable decreases in SOD and CAT activities, along with the reduction in GSH level in the testicular homogenates of contaminated *C. olivieri*, suggest an aberration of the antioxidant system. These enzymes perform key functions in safeguarding cells against oxidative stress impairment; for instance, GSH plays a crucial function in thwarting lipid peroxidation^75^. Furthermore, GPx detoxifies lipid peroxidation by-products, including lipid hydroperoxides using GSH as a reducing agent^76,77^. Therefore, reductions in both GPx and GSH likely contributed to the increased lipid peroxidation and compromised detoxification of lipid hydroperoxides.

It is also believed that GR maintains the supply of GSH^78^, and its lessening activity in contaminated testicular tissue accounts for the observed GSH depletion. In the same sense, GST has a pivotal role in detoxification and neutralization of xenobiotics^79^. Accordingly, its inhibitory activity in beetles from the contaminated site points to a malfunction in the detoxification process. In accordance with our results, a previous report revealed a comparable impairment in the antioxidant and detoxification defense systems of *Ilyocoris cimicoides* because of the pernicious impacts of heavy metals^37^. Furthermore, lessened SOD and CAT activities were perceived in *Lymantria dispar* larvae from contaminated forests following cadmium exposure^80^. Altogether, these malfunctions in antioxidant parameters emphasize the oxidative stress-mediated impairment in the testes of *C. olivieri*.

In light of the abovementioned findings and the observed impairment of redox homeostasis, testicular structural anomalies are anticipated. Earlier studies revealed that exposure to heavy metals and their corresponding nanoparticles could negatively affect spermatogenesis in insects^36,81^. In our study, relevant histopathological characteristics were detectable in the testicular follicles of *C. olivieri* collected from the contaminated location, including cyst wall rupture, damaged parietal cells, vacuolation, and necrosis, which are in line with earlier observations^36,81^. Moreover, ultrastructure examinations of *C. olivieri* testicular tissues from the polluted location revealed pronounced testicular abnormalities, including mitochondrial Nebenkern disintegration. We also observed the disintegration of mitochondrial Nebenkern during the early spermatid stage. These observations concur with previous studies, which manifested Nebenkern membrane disruption and a reduction in Nebenkern matrix density following pollutant exposure^82^. Furthermore, damage to spermatozoal mitochondria could impede ATP production, which is indispensable for sustaining sperm motility^83^. Moreover, spermatozoa deformities were noticed, including abnormal chromatin condensation within the heads of the spermatozoa and malformed acrosomes. Similar malformations were reported in the testis of *Rhynchophorus ferrugineus* (Coleoptera: Curculionidae) exposed to Ivermectin^81^. A previous report highlighted the increasing interest in utilizing various insects in many parts of the world as a sustainable and environmentally friendly source of protein and other essential nutrients^84^. However, our study indicates that the accumulation of hazardous heavy metals in the insect tissues may pose a potential threat to public health.

Collectively, this study emphasizes that heavy metals released from industrial operations, particularly pesticide industries, at the contaminated site can accumulate in testicular tissues and may compromise the reproductive capacity of exposed organisms.

## Conclusion

In conclusion, our study demonstrated the significant utility of *C. olivieri* as a biological indicator for monitoring heavy metal pollution in Kafr El-Zayat, Al Gharbiya Governorate, Egypt. These findings highlight that industrial pesticide operations release hazardous heavy metals, including Ni, Cd, and Cr, which accumulate in *C. olivieri* testicular tissues. These heavy metals compromised the antioxidant and detoxification systems in testes, as evidenced by substantial inhibition of oxidative defense enzymes, including SOD, CAT, GST, GPx, GSH, and GR associated with elevated level of lipid peroxidation and protein carbonyl concertation in the tissues. Moreover, severe structural anomalies were perceived in *C. olivieri* testicular tissues from the contaminated region, including mitochondrial and nuclear damage, deformed spermatogenic elements, and obvious signs of tissue necrosis, implying their correlation with pathophysiological properties. These results underscore the potential ecological and reproductive risks caused by heavy metal contamination to local fauna. Therefore, this study highlights the value of utilizing cost-effective and reliable biomarkers as early warning indicators of various environmental stressors, particularly heavy metals originating from industrial operations. This could significantly contribute to designing effective and judicious strategies to mitigate heavy metal pollution in industrial regions, safeguarding ecosystem fauna.

However, this study has some limitations since it focused on only one bioindicator species and one organ, which may not provide full information of environmental effects across different taxa or tissues. Therefore, future studies should be conducted across multiple species and additional organs to profoundly understand the adverse effects of these heavy metals, which may differ based on each organism and organ’s sensitivity. Moreover, integrating soil and water assessments alongside biological markers could provide a more comprehensive evaluation of heavy-metal contamination and its ecological consequences.

## Data Availability

The datasets used and/or analyzed during the current study are available from the corresponding author on reasonable request.
